# Preconditioning with rHMGB1 ameliorates lung ischemia–reperfusion injury by inhibiting alveolar macrophage pyroptosis via the Keap1/Nrf2/HO-1 signaling pathway

**DOI:** 10.1186/s12967-020-02467-w

**Published:** 2020-08-05

**Authors:** Lin Fei, Xiao Jingyuan, Liang Fangte, Dai Huijun, Ye Liu, Jing Ren, Lin Jinyuan, Pan Linghui

**Affiliations:** Department of Anesthesiology, Guangxi Medical University Cancer Hospital, Nanning, China

**Keywords:** Lung ischemia–reperfusion injury, Recombinant HMGB1, Alveolar macrophage, Pyroptosis, Oxidative stress, Keap1/Nrf2/HO-1 pathway

## Abstract

**Background:**

Lung ischemia–reperfusion injury (LIRI) is a complex pathophysiological process that can lead to poor patient outcomes. Inflammasome-dependent macrophage pyroptosis contributes to organ damage caused by ischemia/reperfusion injury. Oxidative stress and antioxidant enzymes also play an important role in LIRI. In this study, we conducted experiments to investigate whether and how preconditioning with rHMGB1 could ameliorate LIRI in a mouse model.

**Methods:**

Adult male BALB/c mice were anesthetized, the left hilus pulmonis was clamped, and reperfusion was performed. rHMGB1 was administered via intraperitoneal injection before anesthesia, and brusatol was given intraperitoneally every other day before surgery. We measured pathohistological lung tissue damage, wet/dry mass ratios of pulmonary tissue, and levels of inflammatory mediators to assess the extent of lung injury. Alveolar macrophage pyroptosis was evaluated by measuring release of lactate dehydrogenase, caspase-1 expression was assessed using flow cytometry, and gasdermin-D expression was analyzed using immunofluorescent staining. Levels of oxidative stress markers and antioxidant enzymes were also analyzed.

**Results:**

Preconditioning with rHMGB1 significantly ameliorated lung injury induced by ischemia–reperfusion, based on measurements of morphology, wet/dry mass ratios, as well as expression of IL-1β, IL-6, NF-κB, and HMGB1 in lung tissues. It also alleviated alveolar macrophage pyroptosis, reduced oxidative stress and restored the activity of antioxidant enzymes. These beneficial effects were mediated at least in part by the Keap1/Nrf2/HO-1 pathway, since they were reversed by the pathway inhibitor brusatol.

**Conclusions:**

Preconditioning with rHMGB1 may protect against LIRI by suppressing alveolar macrophage pyroptosis. This appears to involve reduction of oxidative stress and promotion of antioxidant enzyme activity via the Keap1/Nrf2/HO-1 pathway.

## Introduction

Lung ischemia–reperfusion injury (LIRI) is a complex pathophysiological process that occurs as a result of various clinical conditions, such as cardiac arrest, trauma, pulmonary thrombosis, lung transplantation, and cardiopulmonary bypass surgery [1, 2]. Respiration failure induced by LIRI is associated with poor patient outcomes and is an important risk factor for acute lung injury (ALI) or acute respiratory distress syndrome (ARDS) [3]. However, the molecular mechanism of LIRI remains unclear, and effective methods to prevent and treat LIRI are still lacking.

Sterile inflammation, occurring as a result of the innate immune response, can play an important role in tissue damage after organ ischemia/reperfusion (I/R) [4]. The magnitude of such inflammation is dependent on the activation of pattern recognition receptors. Alveolar macrophages (AMs) are the primary resident immune cells found in the alveoli of the lungs. They play a pivotal role in the innate immune response and can be activated when pattern recognition receptors on their surface recognize damage-associated molecular patterns (DAMPs) [5, 6]. During the early stages of LIRI, the function of resident AMs is to control inflammation. When the host response cannot re-establish homeostasis, inflammatory responses can expand uncontrollably and progress to ALI and/or ARDS [7, 8].

Pyroptosis refers to programmed cell death that accompanies the release of inflammatory factors [9]. It is mediated by gasdermin-D (GSDMD), which must first be cleaved via the canonical caspase-1 pathway or the noncanonical caspase-11 pathway [10–12]. Recent studies have shown that inflammasome-dependent macrophage pyroptosis can contribute to organ damage caused by I/R injury [13, 14]. How AM pyroptosis in LIRI is activated or regulated remains poorly understood.

High-mobility group box 1 (HMGB1) protein is a damage signal closely associated with factors that can initiate the innate inflammatory response [15, 16]. Studies suggest that preconditioning with recombinant HMGB1 (rHMGB1) can provide protection against myocardial, kidney, and cerebral I/R injury [17–19]. In addition, HMGB1 can activate production reactive oxygen species and induce oxidative stress [20, 21]. Oxidative stress tends to trigger the accumulation of excess inflammatory factors, which leads to a cascade of inflammatory responses, resulting in severe lung tissue damage [22, 23]. Therefore, preconditioning with rHMGB1 to protect the lung against oxidative stress could be feasible technique to reduce the effects of LIRI.

The Keap1/Nrf2/HO-1 signal pathway is closely related to oxidative and anti-oxidative processes [24, 25]. Typically, NF-E2-related factor-2 (Nrf2) and Kelch-like ECH associating protein 1 (Keap1) bind together. However, when stimulated, Nrf2 detaches from Keap1 and translocates into the nucleus, where it activates the production of heme-oxygenase 1 (HO-1). Activation of the Keap1/Nrf2/HO-1 pathway may protect against oxidative stress and apoptosis under various pathological conditions, including organ I/R injury. Recent study further showed that suppressing pyroptosis via the activation of the Nrf2/HO-1 pathway could ameliorate renal I/R injury [26]. Therefore, this study aimed to determine whether preconditioning with rHMGB1 can ameliorate LIRI by inhibiting AM pyroptosis via the Keap1/Nrf2/HO-1 signal pathway.

## Materials and methods

### Animals

We used adult male BALB/c mice (30 ± 5 g; Animal Center, Guangxi Medical University, China). The animal protocol was approved by the Institutional Animal Care and Use Committee of Guangxi Medical University (Nanning, China), and all experiments were conducted in compliance with the Committee’s guidelines.

### Mouse model of LIRI

The LIRI model was induced in mice as previously reported [8]. Animals were anesthetized using pentobarbital (50 mg/kg) via intraperitoneal injection, then mechanically ventilated using a small-animal ventilator (RSP1002-type) at 80 breaths/min and an inspiratory/expiratory ratio of 1:1. The tidal volume was set at 10 ml/kg and the fraction of inspired oxygen was 100%. Animals were closely monitored for lung collapse and expansion.

The mice in the I/R group underwent thoracotomy, followed by 60-min clamping of the left hilus pulmonis including the pulmonary artery, vein, and bronchi). Reperfusion was performed for 120 min, then the thoracic incision was closed. After 2 h, mice were euthanized by cervical vertebra dislocation, and lower portions of the left lung were removed for analysis. No animals died during the experiments.

### Treatment protocols

Some animals were preconditioned with rHMGB1 (20 µg per mouse; < 0.01 EU of endotoxin per mg; Abcam, USA) via intraperitoneal injection at 2 h before anesthesia. The dosage of rHMGB1 was chosen based on a previous study [18]. Some animals received the Nrf2 antagonist brusatol (0.4 mg/kg; BOC Science, USA) in dimethyl sulfoxide five times intraperitoneally (every other day) starting 10 days before I/R surgery. The usages of brusatol based on previous study [22]. In parallel, vehicle groups were treated with vehicle instead of rHMGB1 or brusatol.

### Hematoxylin and eosin (H&E) staining

Experienced technicians who were blinded to experimental groups used 4% paraformaldehyde to fix the lower portions of the left lung tissues as described [8, 27], then embedded them in paraffin. Sections were sliced (4 microns), dewaxed using hydration and xylene, stained with hematoxylin (5 min), treated with hydrochloric acid ethanol (30 s), and soaked in water (15 min) before staining with eosin (2 min). After conventional dehydration, transparent, and sealing, the sections were analyzed as described below.

### Microscopy and histology scoring of lung injury

Slices stained with hematoxylin–eosin were observed using light microscopy [8]. For each mouse, 10 fields were assessed at 200× magnification. An experienced technician who was blinded to group allocation scored the level of lung injury based on three criteria: aggregation or infiltration of inflammatory cells in vessel walls or air spaces [1 point = only wall, 2 = rare cells in air space, 3 = intermediate, and 4 = severe (air space congested)], hyaline membrane formation and interstitial congestion in the lung [1 point = normal lung, 2 = moderate (> 25% of lung section), 3 = intermediate (25–50% of lung section), and 4 = severe (> 50% of lung section)] and presence (1) or absence (0) of hemorrhage. The scores for each criterion were summed to obtain the score for each animal.

### Estimation of wet/dry mass ratio

Pulmonary wet/dry mass ratios were measured as an index of pulmonary edema and congestion [8]. After the mice were euthanized, the lower portions of the left lung were immediately weighed, dried at 60 °C for 24 h, and weighed again.

### Collection of bronchoalveolar lavage fluid and AMs

AMs were isolated as described [28]. Briefly, the lungs were flushed once with cold Dulbecco’s phosphate-buffered saline (5 ml; Gibco BRL, Grand Island, USA) through the cannulated trachea and subsequently flushed eight times with PBS (10 ml) to collect bronchoalveolar lavage fluid (BALF). BALF was then centrifuged at 400*g* for 10 min at 4 °C to remove residual erythrocytes and resuspended in Dulbecco’s modified Eagle medium (DMEM; Gibco). Cells (1 × 10^6^) were counted, transferred to 24-well culture plates (BD, Franklin Lakes, NJ, USA) and incubated for 60 min at 37 °C in a 5% CO_2_ atmosphere. Nonadherent cells were removed by carefully washing with DMEM. Viability of the AMs was evaluated using a 0.2% trypan blue exclusion assay, and their purity was estimated using Ritz-Giemsa staining. Finally, the AMs were counted using a hemocytometer and used in experiments.

### LDH cytotoxicity assay

Lactate dehydrogenase (LDH) levels in the culture supernatant were assessed using the LDH Cytotoxicity Assay Kit (Promega, USA) according to the manufacturer’s instructions. The percentage of total LDH was calculated. The experiment was performed three times.

### Flow cytometry of AMs

AMs from BALF were aliquoted into fluorescence-activated cell sorting tubes at densities of up to 1 × 10^6^ cells per 100 μl, then blocked with immunoglobulin G (1 μg IgG/10^6^ cells) for 15 min at room temperature. The cells were stained with propidium iodide (1:500; Immuno Chemistry Technology, USA), the fluorescent inhibitor of active caspase-1 called FAM-YVAD-FMK (1:500; Immuno Chemistry Technology), and F4/80 antibody (1:500; eBioscience, USA). The samples were incubated in the dark with conjugated antibody (5 μl/10^6^ cells) for 30 min at room temperature. Cells were washed twice using the flow cytometry staining buffer, then resuspended in flow cytometry staining buffer (400 µl) for analysis. Isotype control antibody (Immuno Chemistry Technology) was used as a negative control.

To identify AM pyroptosis, gating was based on F4/80-positive cells, allowing analysis of fluorescently labeled active caspase-1 (FLICA) and propidium iodide. In this approach, F4/80 + FLICA + PI + cells appeared in the upper right quadrant of the FLICA-PI plot and were considered to be pyroptotic AMs [29]. Flow cytometry was conducted using an LSR2 flow cytometer (BD Biosciences), and raw data were analyzed using FlowJo software (TreeStar Corporation, USA).

### Measurement of oxidative stress and anti-oxidant enzymes

We measured levels of the products of oxidative stress [reactive oxygen species (ROS), malondialdehyde (MDA), and 15-F2t-Isoprostane], as well as levels of the antioxidant enzymes superoxide dismutase (SOD), glutathione peroxidase (GSH-PX), and catalase (CAT) in lung tissue. Tissues were homogenized in 5 volumes of RIPA buffer and the supernatants were collected after centrifugation at 2000 rpm for 10 min at 4 °C [22]. The activity of ROS, MDA, SOD, GSH-PX, and CAT were measured using assay kits based on the manufacturer’s instructions (Nanjing Jiancheng Bioengineering Institute, China). Free 15-F2t-isoprostane was measured using an enzyme immunoassay kit (Cayman Chemical, USA). The absorbance from the enzymatic reaction was detected at 412 nm, and values were converted to pg per g of total protein in wet tissue homogenates [26].

### Western blot analysis

Left lung tissues or isolated AMs were homogenized in RIPA buffer (Thermo Scientific, USA) containing a protease inhibitor cocktail (Sigma, USA) and a phosphatase inhibitor cocktail (Roche Applied Science, USA) as described [8, 27]. Homogenates were centrifuged at 13,000 rpm for 20 min at 4 °C, and the supernatant was collected as total protein. Cytoplasmic and nuclear proteins were extracted using NE-PER nuclear and cytoplasmic extraction reagents (Pierce Biotechnology, USA) according to the manufacturer’s instructions. Protein concentration was estimated using a BCA assay (Pierce Biotechnology).

Proteins were separated on a polyacrylamide gel (20 μg per lane) and then transferred onto a polyvinylidene difluoride membrane. The membranes were incubated overnight at 4 °C with primary antibodies against Keap1 (1:200; Santa Cruz, CA, USA), Nrf2 (1:200; Santa Cruz), HO-1 (1:200; Santa Cruz), HMGB1 (1:1000; rabbit polyclonal, Abcam), β-actin (1:5000; mouse monoclonal, Abcam), and lamin A (1:1000; rabbit polyclonal, Abcam). Protein bands were visualized using enhanced chemiluminescence (Pierce, USA), and intensities were normalized to those of β-actin or lamin A. The results obtained under different experimental conditions were normalized to the mean values of the corresponding control.

### Immunofluorescent staining

The expression of GSDMD in isolated AMs was estimated using an immunofluorometric assay. AMs (1 × 10^6^) were cultured on glass coverslips for 24 h, fixed in 4% paraformaldehyde for at least 10 min, and washed three times with PBS. After permeabilization with 0.5% Triton X-100, cells were blocked with 5% BSA (60 min) and incubated overnight on a shaker at 4 °C with rabbit polyclonal anti-GSDMD antibody (1:200; catalog no. NBP2-33422, Novus Biologicals, USA). The sections were then incubated in the dark at room temperature for 1 h with donkey anti-goat IgG antibody conjugated with Alexa Fluor 488 (1:200; Invitrogen, USA). Finally, cell nuclei were stained using 4′,6-diamidino-2-phenylindole (DAPI; Solarbio, China), then viewed under a fluorescence microscope (Olympus BX51).

Image quantification was performed on three randomly selected, non-overlapping fields per section (three sections per mouse) at 100× magnification using an Olympus DP70 microscope (Tokyo, Japan), and the number of pixels per image with an intensity above a predetermined threshold level was quantified using ImageJ software (version 1.47n; National Institutes of Health, Bethesda, MD, USA). The immunoreactivity was expressed as the percentage of total pixels of the imaged field that had an intensity above the threshold level. All quantitative analyses were performed in a blinded manner.

### Enzyme-linked immunosorbent assay

ELISA kits for measuring mouse interleukin-1β (IL-1β; R&D Systems, USA), interleukin-6 (IL-6; R&D Systems, USA), and nuclear factor kappa-B (NF-κB; Abcam, USA) were used to quantify the contents of these cytokines in lung tissues according to the manufacturers’ instructions. Levels of IL-1β, IL-6, and NF-κB in lung tissues were standardized to total protein content.

### Statistical analysis

Continuous data were reported as mean ± SE. Normally distributed data were analyzed using one-way analysis of variance followed by the Tukey test, and skewed data were analyzed using one-way analysis of variance of ranks, followed by the Tukey test. Differences were considered statistically significant when two-tailed p < 0.05. All statistical analyses were performed using SigmaStat (Systat Software, Point Richmond, CA, USA).

## Results

### rHMGB1 preconditioning can ameliorate the inflammatory response and lung tissue damage induced by I/R

**Fig. 1:**
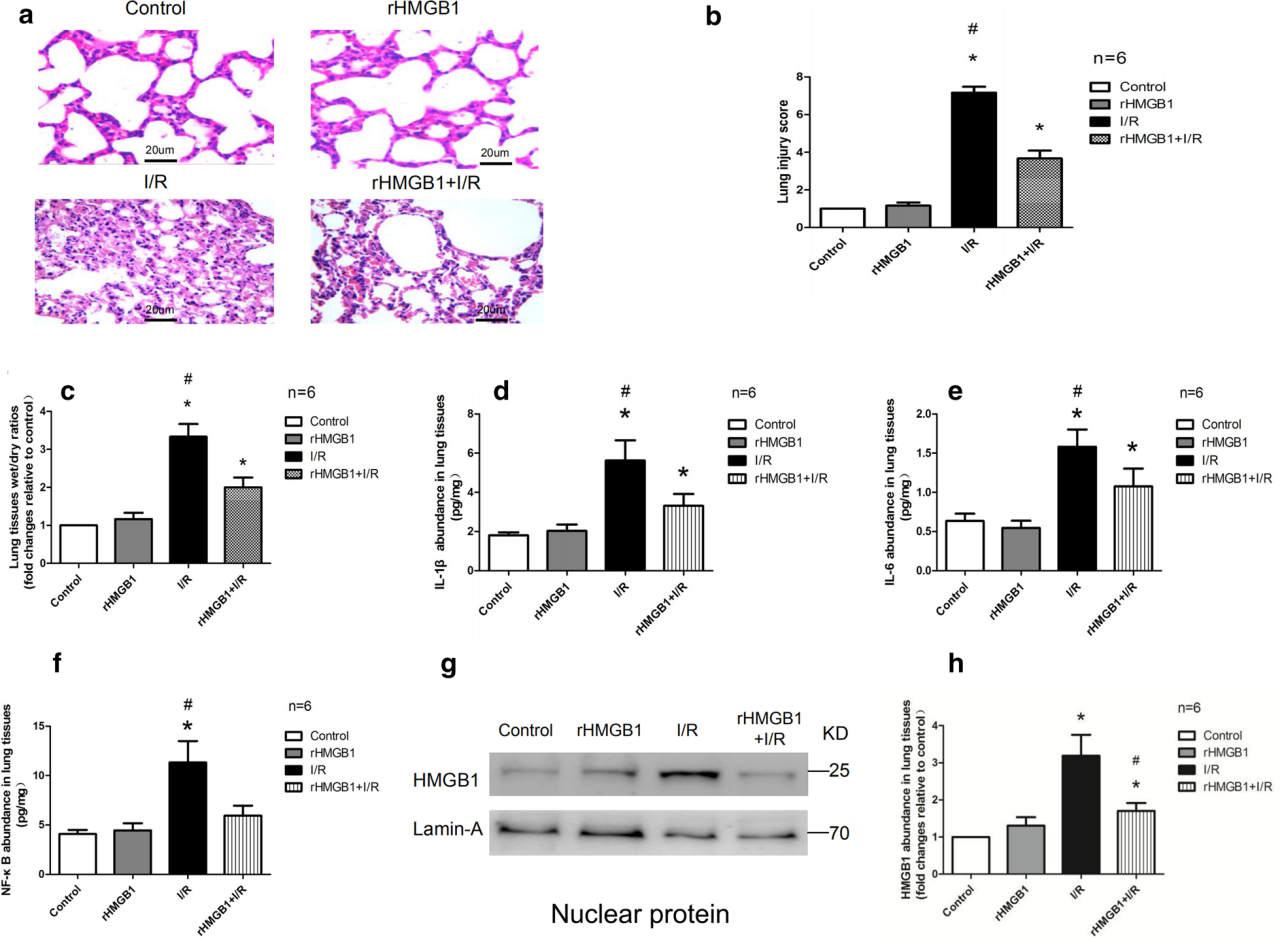
rHMGB1 preconditioning ameliorates tissue damage, inflammatory response, and HMGB1 expression induced by lung I/R injury. **a** Morphological changes in control, rHMGB1, I/R, and rHMGB1+ I/R groups observed using H&E staining. Magnification, ×200. **b** Lung injury scoring. **c** Wet/dry ratios in lung tissues. **d** IL-1β abundance in lung tissues. **e** IL-6 abundance in lung tissues. **f** NF-κB abundance in lung tissues. **g** Representative western blot images of HMGB1 in nuclear protein. **h** HMGB1 abundance in nuclear protein. (*p < 0.05 vs control group, ^#^p < 0.05 vs I/R and rHMGB1+ I/R group)

To confirm whether rHMGB1 preconditioning plays a key role in LIRI, rHMGB1 was intraperitoneally injected at 2 h before surgery. The control and the rHMGB1 group showed an intact alveolar structure with minor inflammatory cell infiltration (Fig. 1a). The I/R group had significant alveolar wall rupture, neutrophil and erythrocyte infiltration, as well as interstitial edema. These morphological changes were less severe in the rHMGB1 + I/R group. Similar results were observed based on lung injury scores and pulmonary wet/dry ratios (Fig. 1b, c). ELISA showed that levels of IL-1β, IL-6, and NF-κB were obviously higher in the I/R group than in the control and rHMGB1 groups. Levels were lower in the rHMGB1 + I/R group than in the I/R group (Fig. 1d–f). The results of western blot analysis showed that preconditioning with rHMGB1 reduced HMGB1 expression induced by I/R injury (Fig. 1g, h). In fact, no differences were observed between the control and rHMGB1 group. These results suggest that preconditioning with rHMGB1 can ameliorate I/R-induced inflammatory response and lung tissue damage.

### rHMGB1 preconditioning can mitigate AM pyroptosis induced by lung I/R

**Fig. 2:**
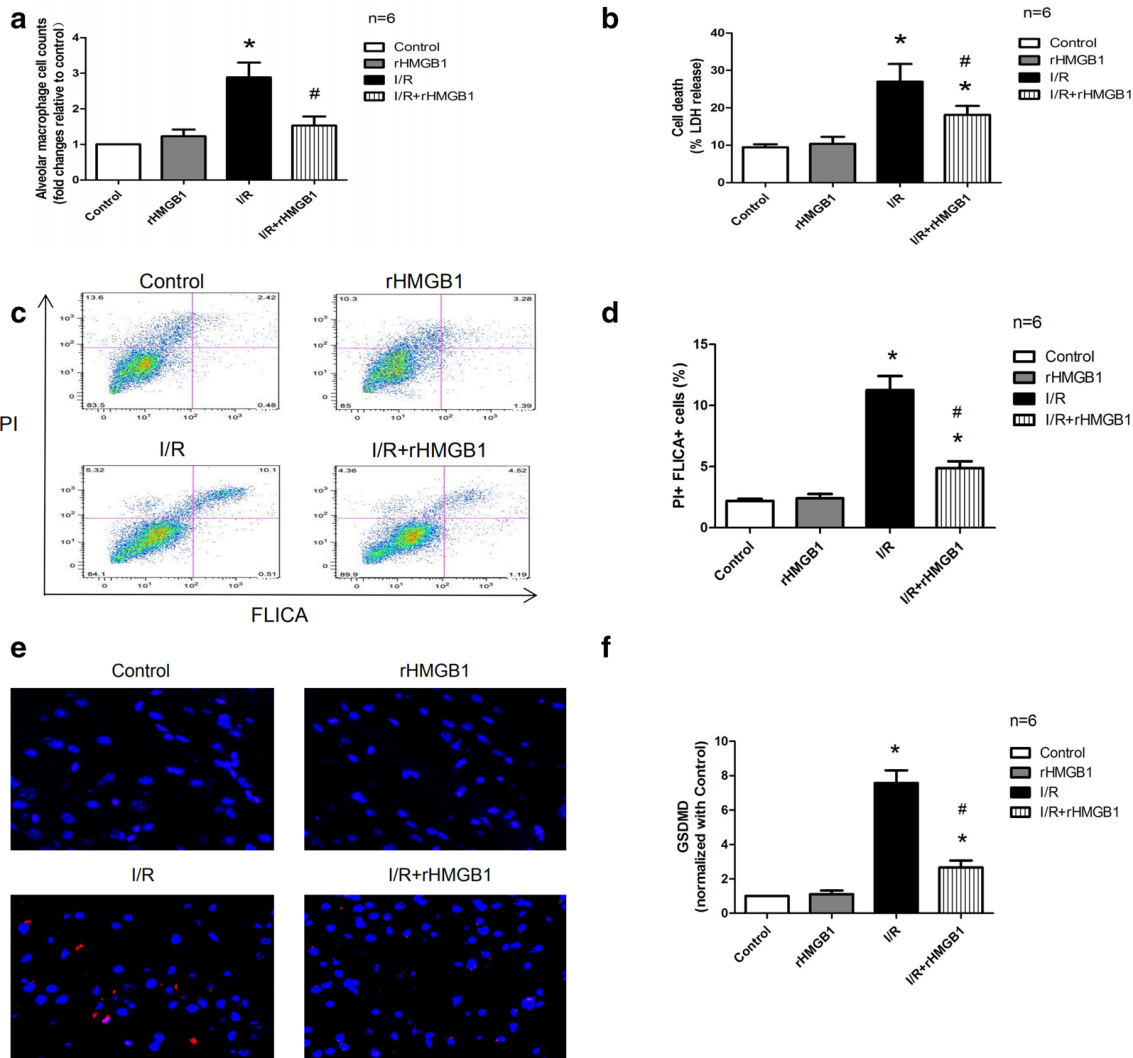
rHMGB1 preconditioning decreases I/R-induced AM pyroptosis. **a** Isolated AM count in BALF. **b** LDH release from isolated AMs in BALF. **c** Representative results from flow cytometry to assess macrophage pyroptosis: F4/80^+^ cells were gated and analyzed for fluorescently labeled active caspase (FLICA) and propidium iodide (PI). **d** Quantitative analysis of F4/80^+^FLICA^+^PI^+^ cells. **e** Representative images of isolated AMs in BALF after immunostaining for GSDMD. **f** GSDMD abundance in isolated AMs. (* p < 0.05, compared to control group, ^#^p < 0.05, comparison between I/R and rHMGB1 + I/R group)

When we counted the number of AMs isolated from BALF, we found a significantly higher number in the I/R group than in the control and rHMGB1 groups. Preconditioning with rHMGB1 significantly reduced the number of AMs induced by I/R (Fig. 2a). Next, we evaluated whether lung I/R injury could induce AM pyroptosis. Distinctive features of pyroptosis include plasma membrane rupture, LDH release, caspase activation, and GSDMD cleavage [11]. LDH release in samples of isolated AMs was markedly higher in the I/R group than in the control and rHMGB1 groups, and rHMGB1 preconditioning led to a significantly smaller increase in LDH release (Fig. 2b). Similar results were obtained in the flow cytometry analysis (Fig. 2c, d). Immunofluorescence staining showed that I/R injury significantly up-regulated GSDMD in isolated AMs, which rHMGB1 preconditioning partially reversed (Fig. 2e, f). These results suggest that rHMGB1 preconditioning can alleviate AM recruitment and pyroptosis induced by lung I/R injury.

### rHMGB1 preconditioning inhibits oxidative stress and increases anti-oxidant enzyme levels in LIRI

**Fig. 3:**
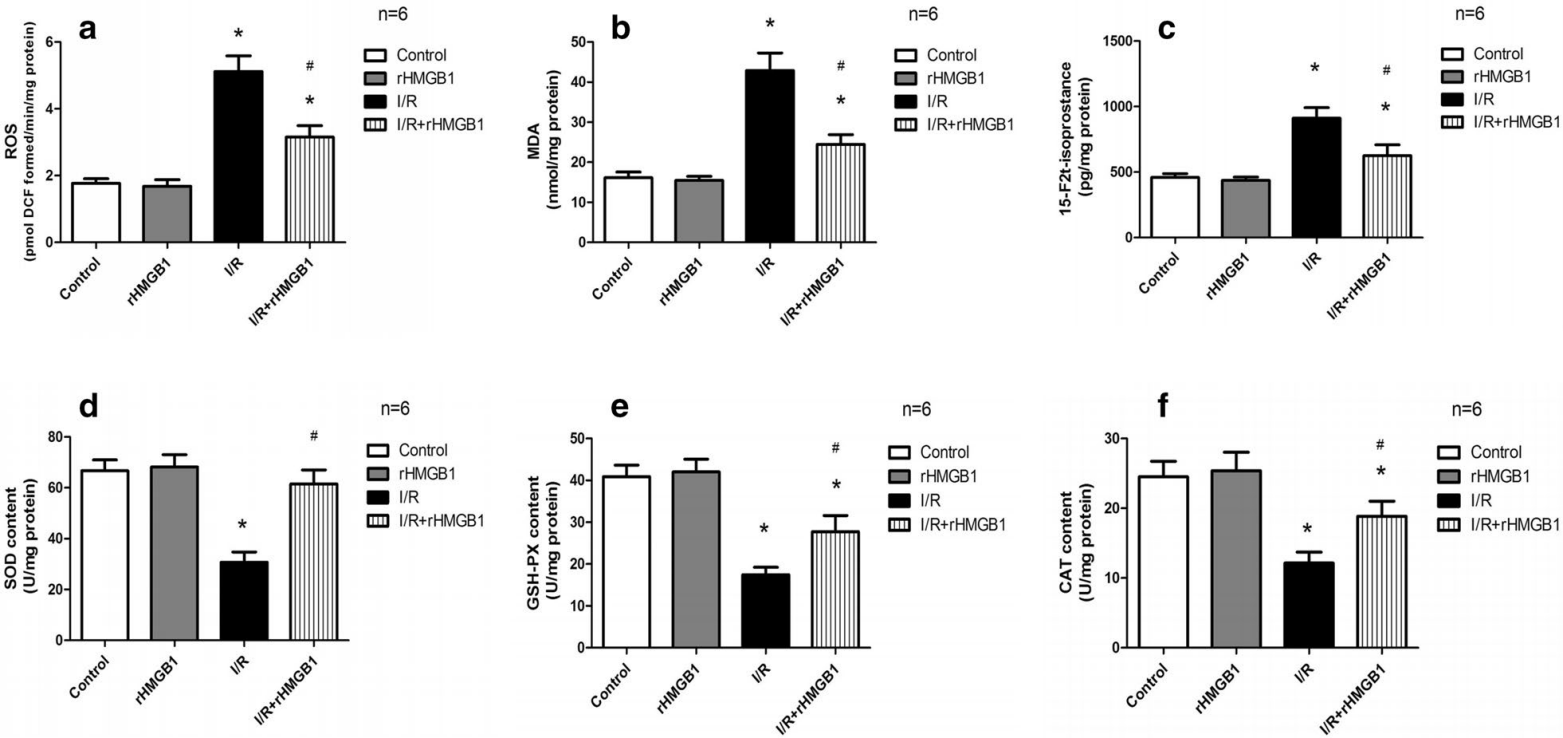
rHMGB1 preconditioning inhibits I/R-induced oxidative stress and restores anti-oxidant enzyme levels. **a** ROS. **b** MDA. **c** 15-F2t-isoprostane. **d** SOD. **e** GSH-PX. **f** CAT. (*p < 0.05, compared to control group, ^#^p < 0.05, comparison between I/R and rHMGB1 + I/R group)

To understand the effects of rHMGB1 on oxidative and anti-oxidative processes during lung I/R injury, the products of oxidative stress (ROS, MDA, 15-F2t-Isoprostane) and antioxidant enzymes (SOD, GSH-PX, CAT) were measured. The levels of ROS, MDA, and 15-F2t-isoprostane were markedly higher in the I/R group than in the control and rHMGB1 groups (Fig. 3a–c). Preconditioning with rHMGB1 led to a significantly smaller increase. Conversely to the levels of oxidation products, the I/R group showed markedly lower activity of anti-oxidant enzymes than the control and rHMGB1 groups (Fig. 3d–f). Preconditioning with rHMGB1 partially restored these levels. These results suggest that pretreatment with rHMGB1 can inhibit oxidative stress and increase the activity of anti-oxidant enzymes during lung I/R injury.

### rHMGB1 preconditioning can mediate the activity of the Keap1/Nrf2/HO-1 pathway in LIRI

**Fig. 4:**
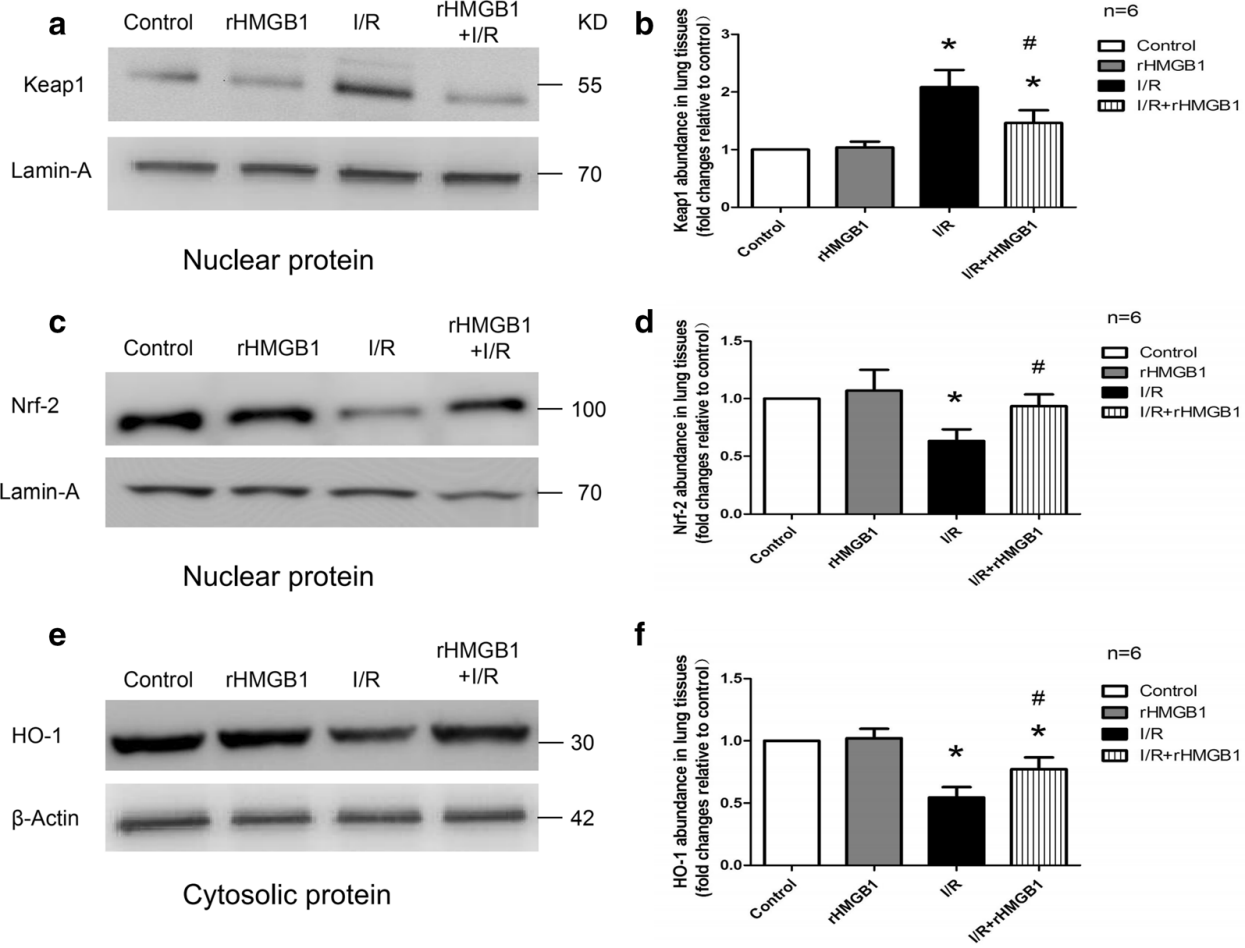
rHMGB1 preconditioning mediates the activity of the Keap1/Nrf2/HO-1 pathway in a mouse model of lung I/R. **a** Representative western blot images of nuclear Keap1 in lung tissues. **b** Nuclear Keap1 levels in lung tissues. **c** Representative western blot images of nuclear Nrf2 in lung tissues. **d** Nuclear Nrf2 expression levels in lung tissues. **e** Representative western blot images of cytosolic HO-1 in lung tissues. **f** Cytosolic HO-1 expression levels in lung tissues. (*p < 0.05, compared to control group, ^#^p < 0.05, comparison between I/R and rHMGB1 + I/R group)

The Keap1/Nrf2/HO-1 pathway plays an important role in oxidative and anti-oxidative processes. Western blots were used to analyze expression levels of the nuclear proteins (Keap1, Nrf2) and the cytosolic protein (HO-1). Lung I/R injury significantly up-regulated Keap1 and down-regulated Nrf2 and HO-1, which rHMGB1 preconditioning partially reversed (Fig. 4). These results suggest that pretreatment with rHMGB1 can mediate the activity of the Keap1/Nrf2/HO-1 pathway in LIRI.

### Inhibition of the Keap1/Nrf2/HO-1 pathway blocks the protective effects of rHMGB1 preconditioning in LIRI

**Fig. 5:**
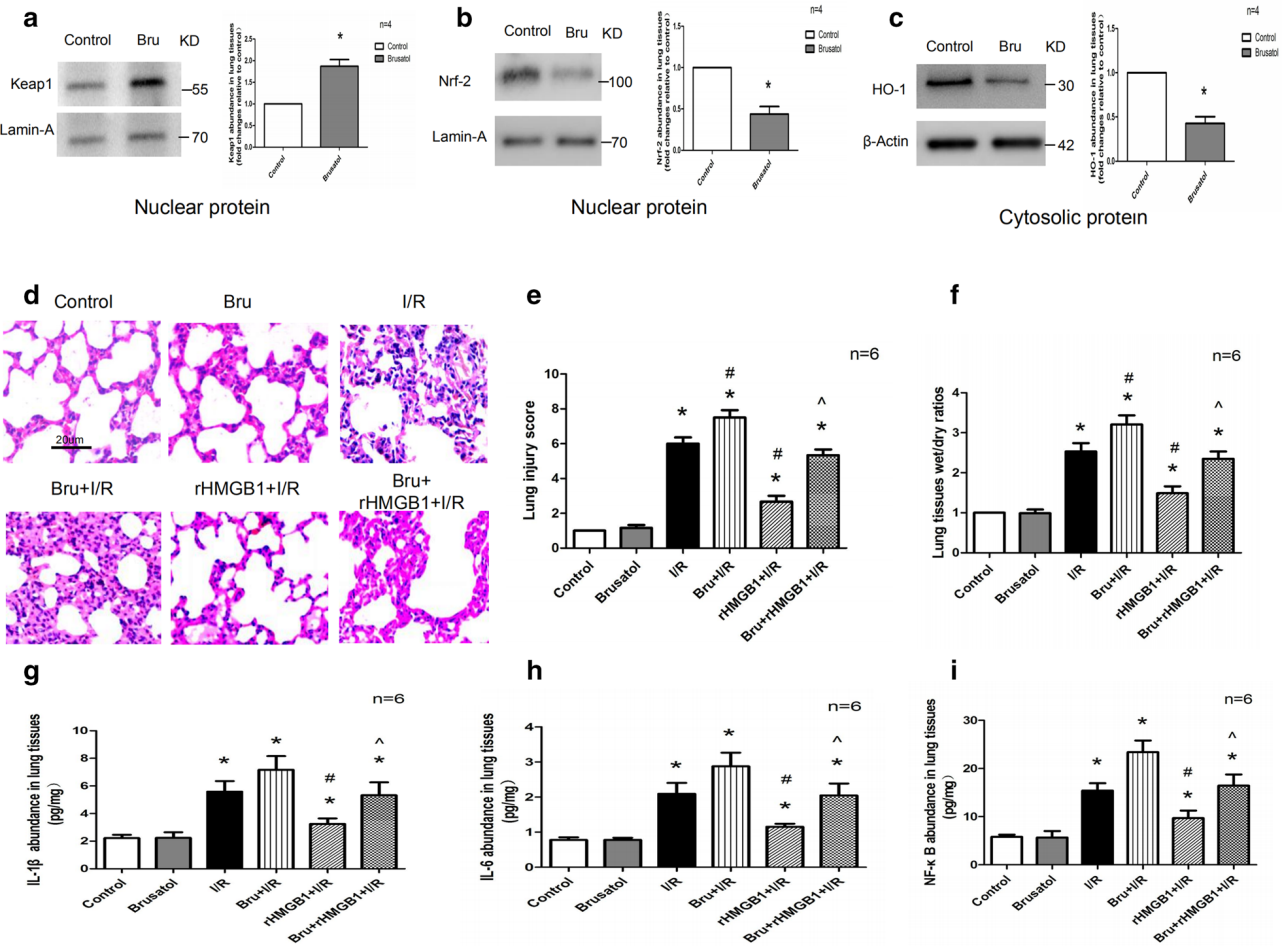
Keap1/Nrf2/HO-1 pathway inhibition alleviates the protective effects of rHMGB1 preconditioning in LIRI. **a** Western blot of nuclear Keap1 in lung tissues. **b** Western blot of nuclear Nrf2 in lung tissues. **c** Western blot of cytosolic HO-1 in lung tissues. **d** Morphological changes across groups observed using H&E staining. Magnification, ×200. **e** Lung injury scoring. **f** Wet/dry ratios in lung tissues. **g** IL-1β abundance in lung tissues. **h** IL-6 abundance in lung tissues. **i** NF-κB abundance in lung tissues. (*p < 0.05, compared to control group, ^#^p < 0.05, compared to I/R group, ^^^p < 0.05, comparison between rHMGB1 + I/R and brusatol + rHMGB1 + I/R group)

The experiment described above suggested that the protective effects of rHMGB1 in LIRI depend on the Keap1/Nrf2/HO-1 pathway. To confirm this, the pathway inhibitor brusatol was administered, which significantly up-regulated Keap1 and down-regulated Nrf2 and HO-1 (Fig. 5a–c) as expected. Brusatol exacerbated the alveolar destruction, inflammatory cell and erythrocyte infiltration, and alveoli septum thickening induced by I/R (Fig. 5d). Damage to lung tissues was worse in the brusatol + rHMGB1 + I/R group than in the rHMGB1 + I/R. Similar results were observed based on lung injury score and pulmonary wet/dry ratio. Brusatol further increased the levels of IL-1β, IL-6, and NF-κB observed after I/R (Fig. 5g–i). Levels of these cytokines were higher in the brusatol + rHMGB1 + I/R group than in the rHMGB1 + I/R group. These results suggest that brusatol blocks the protective effects of rHMGB1 in LIRI.

### Inhibition of the Keap1/Nrf2/HO-1 pathway blocks the anti-oxidant effects of rHMGB1 in LIRI

**Fig. 6:**
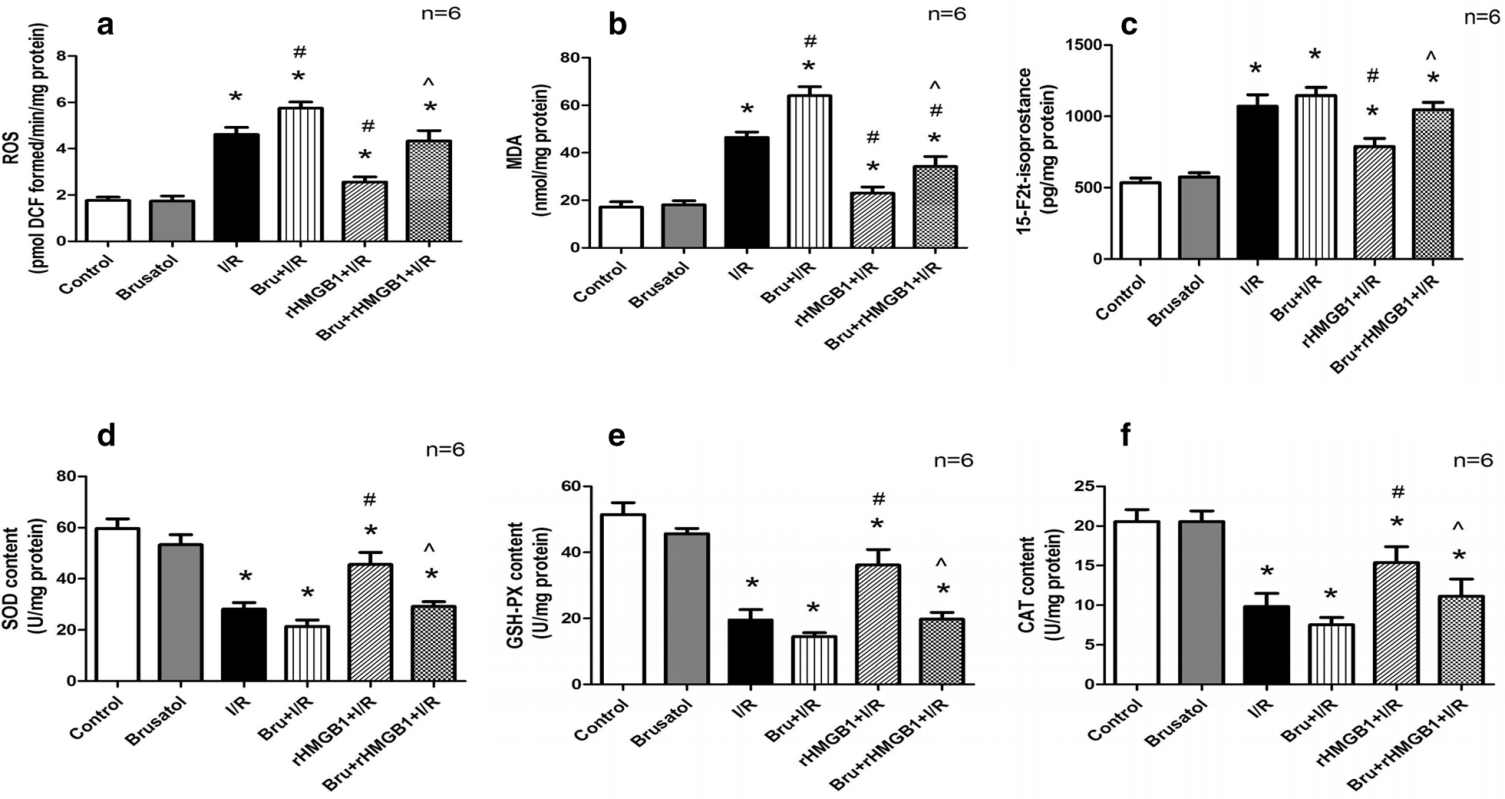
Keap1/Nrf2/HO-1 pathway inhibition can suppress the anti-oxidant effects of rHMGB1 preconditioning in LIRI. **a** ROS. **b** MDA. **c** 15-F2t-isoprostane. **d** SOD. **e** GSH-PX. **f** CAT. (*p < 0.05, compared to control group, ^#^p < 0.05, compared to I/R group, ^^^p < 0.05, comparison between rHMGB1 + I/R and brusatol + rHMGB1 + I/R group)

I/R increased levels of oxidative stress products (ROS, MDA, and 15-F2t-isoprostane). While rHMGB1 markedly alleviated oxidative stress, brusatol had the opposite effect. Levels of ROS, MDA, and 15-F2t-isoprostane were higher in the brusatol + rHMGB1 + I/R group than in the rHMGB1 + I/R group (Fig. 6a–c). Conversely, I/R reduced levels of the anti-oxidant enzymes SOD, GSH-PX, and CAT, which rHMGB1 pretreatment reversed. SOD, GSH-PX, and CAT activity was lower in the brusatol + rHMGB1 + I/R group than in the rHMGB1 + I/R group (Fig. 6d–f). These results demonstrate that inhibition of the Keap1/Nrf2/HO-1 pathway blocks the anti-oxidant effects of rHMGB1 pretreatment in LIRI.

### rHMGB1 preconditioning acts via the Keap1/Nrf2/HO-1 pathway to inhibit AM pyroptosis in LIRI

**Fig. 7:**
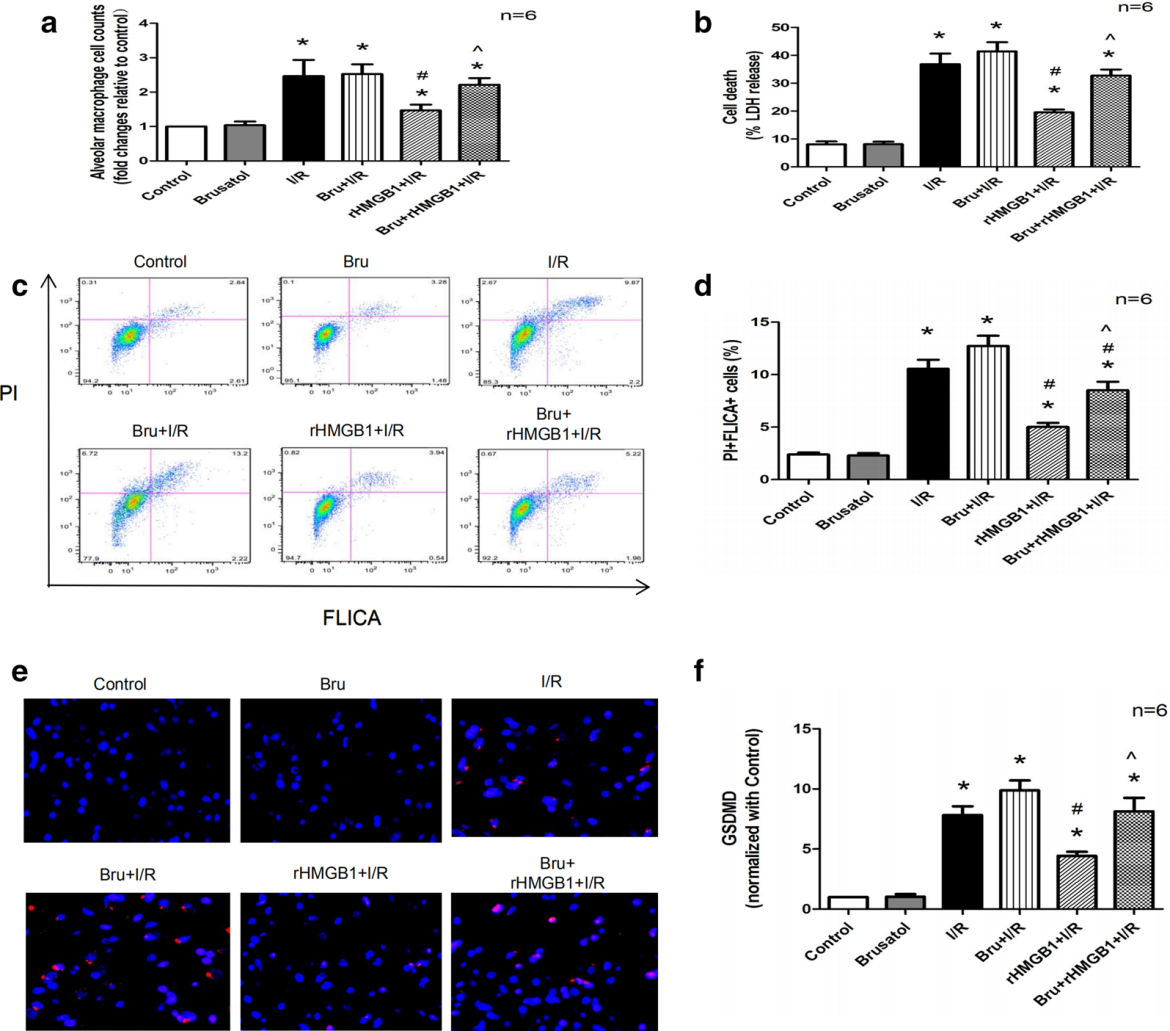
rHMGB1 preconditioning inhibits AM pyroptosis via the Keap1/Nrf2/HO-1 pathway in LIRI. **a** Isolated AM counts in BALF. **b** LDH release from isolated AMs in BALF. **c** Representative results of flow cytometry assessing macrophage pyroptosis: F4/80^+^ cells were gated and analyzed for fluorescently labeled active caspase (FLICA) and propidium iodide (PI). **d** Quantitative analysis of F4/80^+^FLICA^+^PI^+^ cells. **e** Representative immunolabelling images for GSDMD protein from isolated AMs in BALF. **f** GSDMD levels in isolated AMs. (*p < 0.05, compared to control group, ^#^p < 0.05, compared to I/R group, ^^^p < 0.05, comparison between rHMGB1 + I/R and brusatol + rHMGB1 + I/R group)

The number of AMs isolated from BALF was significantly higher in the I/R group than in the control group. Preconditioning with rHMGB1 led to a smaller increase, while brusatol had the opposite effect (Fig. 7a). Changes in LDH release showed similar trends (Fig. 7b). Flow cytometry showed that rHMGB1 preconditioning significantly reduced AM pyroptosis induced by lung I/R injury, which brusatol partially reversed (Fig. 7c, d). Immunofluorescence staining of isolated AMs showed that rHMGB1 preconditioning also led to weaker GSDMD up-regulation in response to I/R injury, which brusatol partially reversed (Fig. 7e, f). These results suggest that inhibition of the Keap1/Nrf2/HO-1 pathway blocks the anti-pyroptotic effects of rHMGB1 preconditioning in LIRI.

## Discussion

In this study, we evaluated the effects of lung I/R injury on mouse models after preconditioning with rHMGB1. Our results suggest that rHMGB1 preconditioning can provide protection against lung damage induced by lung I/R injury. These findings are consistent with previous studies conducted on I/R injury in different organs [17–19, 30], indicating the potential clinical significance of rHMGB1 preconditioning as a preventive strategy to prevent and treat LIRI.

Preconditioning aims to cause minor injury in order to stimulate the body to defend itself against a subsequent major injury. Activated macrophages as well as necrotic or damaged cells secrete the cytokine HMGB1, which induces local and systemic inflammatory responses [31]. The present study shows that delivery of exogenous rHMGB1 can mitigate damage caused by LIRI in a mouse model. We further implicated the Keap1/Nrf2/HO-1 pathway in the beneficial effects of preconditioning.

Macrophages are resident immune cells that play a vital role in the innate immune response of the body. AMs are the primary resident cells within lung and can be activated by DAMPs during LIRI progression. Macrophage pyroptosis plays an important role in the body’s innate immune defense against intracellular bacteria or DAMPs, but uncontrolled pyroptosis can lead to tissue damage. Inflammasomes promote the activation of inflammatory caspases, such as caspases-1, -4, -5, -11, ultimately triggering the cleavage of GSDMD, which in turn induces pyroptosis [9, 10]. Therefore, the process of pyroptosis strikes an unstable balance between protective host-defense responses and harmful explosive inflammatory responses [11, 12]. Targeting pyroptosis in macrophages may be a potential strategy for inhibiting inflammatory response and organ damage [13, 14]. In this study, we found that preconditioning with rHMGB1 mitigated the I/R-induced up-regulation of proteins involved in pyroptosis, ultimately dampening the release of inflammatory factors.

Oxidative stress is another critical factor in the pathophysiology of LIRI [32]. During I/R injury, macrophages, neutrophils, and endothelial cells can produce ROS, which in turn induces release of pro-inflammatory cytokines. Excess ROS generation also triggers lipid peroxidation of cell membranes, leading to cell death. The products of lipid peroxidation, such as MDA and 15-F2t-isoprostane, are significantly increased in animal models of lung I/R injury, which exacerbates the inflammatory response, pulmonary edema, and tissue damage [22, 33]. Anti-oxidant enzymes such as SOD, GSH-PX, and CAT can eliminate reactive free radicals to maintain the balance between oxidative and anti-oxidative stress responses. Suppressing the activity of these anti-oxidant enzymes aggravates inflammatory responses and tissue damage during lung I/R injury [22, 34]. Consistently, we found that lung I/R injury in our mouse model of LIRI increased the levels of products of oxidative stress (ROS, MDA, 15-F2t-Isoprostane) and down-regulated the anti-oxidant enzymes SOD, GSH-PX, and CAT.

HMGB1 can mediate inflammation via multiple ways; it can activate ROS and induce oxidative stress [20, 21]. Interestingly, our results show that preconditioning with rHMGB1 reversed the increased production of oxidative stress markers and the down-regulation of anti-oxidant enzymes during lung I/R injury. Therefore, it is reasonable to conclude that rHMGB1 preconditioning helps protect against LIRI by down-regulating HMGB1 and by inhibiting oxidative stress, AM pyroptosis and release of inflammatory factors during lung I/R injury.

To understand the possible mechanism underlying the protective effect of rHMGB1 preconditioning during LIRI, we assessed the role played by the Keap1/Nrf2/HO-1 signal pathway in suppressing oxidative stress and AM pyroptosis. Nrf2 is a transcription factor that helps mediate anti-oxidant responses to stress [35], and the Keap1/Nrf2/HO-1 pathway is the primary cellular defense against the cytotoxic effects of oxidative stress [25, 36]. Under normal conditions, Nrf2 and Keap1 form a complex, and Keap1 mediates Nrf2 ubiquitination and degradation. After stimulation in response to stress, the Keap1/Nrf2 complex breaks up in the nucleus and Nrf2 translocates to the cytoplasm, where it turns on the HO-1 gene [37]. Activation of the Keap1/Nrf2/HO-1 pathway may protect against oxidative stress and pyroptosis in different organs affected by I/R injury [22, 25, 26]. In this study, we observed that lung I/R injury up-regulated nuclear Keap1 and down-regulated nuclear Nrf2 and cytosolic HO-1. Preconditioning with rHMGB1 inhibited the activity of the Keap1/Nrf2/HO-1 pathway in LIRI.

We confirmed the role of the Keap1/Nrf2/HO-1 pathway in maintaining the protective effects of rHMGB1 during LIRI using the Nrf2 antagonist brusatol. Brusatol possesses various pharmacological properties, such as anticancer, anti-inflammatory, antiprotozoal, and antiphytoviral effects. By blocking the Nrf2 pathway, brusatol selectively reduces the levels of Nrf2 by stimulating its ubiquitination and proteolysis. We found that brusatol blocked the anti-oxidant and anti-pyroptotic effects of rHMGB1 in LIRI. In order to avoid the impact of its biological characteristics on animal models, brusatol group was designed to be compared with the control group, and our results showed that no statistical difference was found between the two groups.

Limitation of this study is that the molecule mechanism of rHMGB1 involved in the regulation of Keap1/Nrf2/HO-1 remains unclear. Further research in which AMs transfected with siRNA of Keap1/Nrf2/HO-1 in oxygen–glucose deprivation/recovery (OGD/R) vitro model needs to be conducted to verify the present study and explore the specific correlation between rHMGB1 and Keap1/Nrf2/HO-1.

## Conclusions

These results provide evidence that rHMGB1 preconditioning can mitigate LIRI by suppressing AM pyroptosis. This preconditioning appears to reduce oxidative stress and increase the activity of anti-oxidant enzymes via the Keap1/Nrf2/HO-1 pathway.

## Data Availability

The datasets used and/or analyzed during the current study are available on reasonable request from the corresponding author.

## Abbreviations

LIRI: Lung ischemia–reperfusion injury
I/R: Ischemia/reperfusion
AMs: Alveolar macrophages
ALI: Acute lung injury
ARDS: Acute respiratory distress syndrome
DAMPs: Damage-associated molecular patterns
GSDMD: Gasdermin-D
HMGB1: High-mobility group box 1
rHMGB1: Recombinant HMGB1
Nrf2: NF-E2-related factor-2
Keap1: Kelch-like ECH associating protein 1
HO-1: Heme-oxygenase 1
BALF: Bronchoalveolar lavage fluid
LDH: Lactate dehydrogenase
ROS: Reactive oxygen species
MDA: Malondialdehyde
SOD: Superoxide dismutase
GSH-PX: Glutathione peroxidase
CAT: Catalase
IL-1β: Interleukin-1β
IL-6: Interleukin-6
NF-κB: Nuclear factor kappa-B
